# Abnormal expression profile of plasma exosomal microRNAs in exclusive electronic cigarette adult users

**DOI:** 10.21203/rs.3.rs-3877316/v1

**Published:** 2024-01-24

**Authors:** Dongmei Li, Zidian Xie, Sadiya Bi Shaikh, Irfan Rahman

**Affiliations:** University of Rochester Medical Center; University of Rochester Medical Center; University of Rochester Medical Center; University of Rochester Medical Center

**Keywords:** Plasma, exosome, microRNAs, PATH, exclusive e-cigarette users, adults, cancer

## Abstract

**Background:**

Exposure to electronic cigarette (e-cigarette) aerosol has been linked to several health concerns, including DNA damage, elevated oxidative stress, the release of inflammatory cytokine, and dysfunctions in epithelial barriers. However, little is known about the effect of exclusive e-cigarette use on expression profiles of exosomal miRNAs, which play critical regulatory roles in many inflammatory responses and disease processes including cancer. We aim to compare the exosomal microRNA expression profile between exclusive e-cigarette users and normal controls without any tobacco product use (non-users).

**Methods:**

Using plasma samples from 15 exclusive e-cigarette users and 15 non-users in the Population Assessment of Tobacco and Health (PATH) Wave 1 study (2013–2014), we examined exosomal microRNAs expression levels through Illumina NextSeq 500/550 sequencing. The differential analyses between exclusive e-cigarette users and non-users were examined using the generalized linear model approach in the *DESeq2* package in R/Bioconductor after adjusting the significant confounding effect from race. Gene enrichment analyses were conducted on target genes regulated by significant microRNAs in the differential analyses. Further, molecular-based techniques using the micro RNA mimics and inhibitors were applied for the validation of the expressions of the micro RNAs *in vitro*.

**Results:**

We identified four microRNAs that have significantly higher expression levels in exclusive e-cigarette users than non-users including hsa-miR-100–5p, hsa-miR-125a-5p, hsa-miR-125b-5p, and hsa-miR-99a-5p. GO enrichment analysis on the target genes regulated by the four microRNAs showed that dysregulation of the four microRNAs in exclusive e-cigarette users involved in multiple cell processes such as protein kinase binding and miRNA metabolic process. KEGG pathway enrichment analysis found the four upregulated miRNAs in exclusive e-cigarette users involved in many cancer pathways such as the non-small cell lung cancer, small cell lung cancer, pancreatic cancer, p53 signaling pathway, Hippo signaling pathway, HIF-1 signaling pathway, and MAPK signaling pathway. Overexpression of miRNA hsa-miR-125b-5p was shown to promote DNA damage in bronchial epithelia cells.

**Conclusions:**

Four plasma exosomal microRNAs involved in cancer development had higher expression levels in exclusive e-cigarette users than non-users, which might indicate a potentially elevated risk of cancer among exclusive e-cigarette users.

## Introduction

An electronic cigarette (e-cigarette) is a device designed to simulate the feeling of traditional cigarette smoking to vaporize a liquid (often flavored) to generate aerosols for inhalation [1]. The e-liquid typically contains propylene glycol and/or vegetable glycerin as well as nicotine and flavoring agents/chemicals [2]. According to the 2023 National Youth Tobacco Survey, 10.0% of high school students and 4.6% of middle school students reported current use of e-cigarettes [3]. E-cigarette delivers nicotine to users, leading to addiction comparable to or even exceeding that of conventional cigarettes [4]. While e-cigarettes appear to reduce or eliminate certain carcinogens found in combustible cigarettes, the toxicological effects of inhaling e-cigarette aerosols containing flavorings remain unclear [5–7].

Animal and cell line studies indicate that e-cigarette aerosols can trigger oxidative stress, cause DNA damage, and induce inflammatory responses in both human lung epithelial cells and mouse lungs [8–13]. Epidemiology studies demonstrate a correlation between e-cigarette use and respiratory symptoms and diseases such as wheezing, asthma, and chronic obstructive pulmonary disease (COPD) [14–18]. Additionally, e-cigarette use is associated with an increased risk of hypertension and cardiovascular diseases, including myocardial infarction, stroke, and coronary artery disease [19–23]. Mental health problems, depressions, and cognitive impairments have also been linked to e-cigarette use [23–26].

Exosomes are small extracellular vesicles (EVs) released by various cell types, including immune and cancer cell, and are present in biofluids such as blood, urine, and other body fluids [27, 28]. Exosomes play a crucial role in mediating cell-to-cell communication and influencing numerous physiological processes [29–32]. Exosomes are enriched with surface proteins, regulatory proteins, mRNAs, microRNAs and long non-coding RNAs [33, 34]. Plasma exosomes, in particular, hold potential as biomarkers for immune dysfunction in cancer [35].

MicroRNAs (miRNAs), a class of small non-coding RNAs consisting of 21–25 nucleotides, play vital regulatory roles in various cellular and biological process, including immune regulation and inflammatory responses [36–39]. miRNAs have been reported to play pivotal roles in the development of chronic inflammatory lung diseases and lung cancers [40–45]. Exosomal miRNAs, aside from revealing novel toxicological subtypes, show promise as potential biomarkers for flavor-related toxicities. Previous research has demonstrated that tobacco consumption modulates the expression levels of miRNAs through signaling pathways such as apoptosis, angiogenesis and inflammatory pathways [46–48]. These pathways play significant roles in the development of various human diseases, including lung disease, cardiovascular disease, liver disease, and cancer [49–51]. Exosomal microRNAs has emerged as diagnostic biomarkers and therapeutic targets for these diseases. Currently, there is a lack of information on the patterns of genome-wide and locus-specific miRNA expression in plasma samples when the subjects are exposed to e-cigarettes.

This study utilized plasma samples obtained from participants in the Population Assessment of Tobacco and Health (PATH) Study Wave 1 (2013–2014), including 15 exclusive e-cigarette users and 15 non-users [52]. Our aim is to identify plasma exosomal miRNAs that are differentially expressed between participants who exclusively use e-cigarettes and participants who don’t use any tobacco products. We seek to understand the functions of identified significant miRNAs in various signaling pathways. The identified plasma exosomal miRNAs and their associated targeted genes will significantly contribute to our understanding of the impact of e-cigarettes on human health. Given the pivotal role miRNAs play in the pathogenesis of numerous diseases, the identified biomarkers hold potential for use as prognostic indicators in disease diagnosis and as therapy targets in future studies.

## Materials and Methods

### Study Participants

The PATH study is a nationally representative longitudinal cohort study initiated in September 2013 with 32,320 adults and 13,651 youth (12 years and older) participants [52]. In addition to collecting epidemiological information on tobacco use behaviors, attitudes, beliefs, exposures, and health, the PATH study includes biospecimens from a subsample of 11,500 adult participants from the Wave 1 study. Blood specimens were exclusively collected from PATH Wave 1 adults and aged-up adults from Wave 1 youth participants.

We obtained plasma samples through the PATH Study Biospecimen Access Program from a cohort of 15 current adult (aged 18 years or older) established exclusive e-cigarette users and 15 adult non-users who were not established any tobacco product users. Current established exclusive e-cig users are defined as individuals who have ever used an e-cigarette, have used regularly, and use every day or some days. Furthermore, these current established exclusive e-cig users do not regularly use any other tobacco products.

The study has been reviewed by the University of Rochester Research Subjects Review Board and was determined as not research involving human subjects. All biospecimens obtained from the PATH biospecimen program were deidentified, and the linkage ID connecting demographic information with the PATH biospecimens were disclosed only after the completion of sequencing experiments on all biospecimens.

### Sample collection

Biospecimens, including urine and blood, are acquired from PATH Study participants who have provided informed consent, following a protocol reviewed and approved by the Westat Institutional Review Board (IRB) using validated PATH Study standard operating procedures (SOPs). The collected biospecimens were transported from the field to the PATH Study biorepository and subsequently processed into aliquots. Following approval from the PATH Study Biospecimen Access program, we received 30 plasma aliquots (200ul each) via mail in October 2021.

### Exosome isolation and RNA extraction

Exosome RNA extraction was performed using Norgen’s Exosomal RNA Isolation Kit (Cat. 58000), and the subsequent preparation of the next-generation sequencing library was conducted using Norgen Biotek’s Small RNA Library Prep Kit for Illumina (Cat. 63620).

### Next-generation sequencing

The next-generation sequencing analysis was carried out using the Illumina NextSeq 500 sequencing platform, employing the NextSeq 500/550 High Output Kit v2 (51 Cycles with a 75-Cycle Kit) as the sequencing platform reagent.

### Statistical analysis

Unique participant IDs were used to establish links between PATH demographic data and the sequencing outcomes of plasma exosomal miRNAs. Fisher’s exact tests were used to identify potential confounding variables for the e-cigarette user group. miRNAs with a total count below 10 across all samples were excluded from subsequent analyses. The DESeq2 package in R/Bioconductor was used to identify statistically significant miRNAs differentially expressed between exclusive e-cigarette users and non-users, while accounting for significant confounding variable. miRNAs with a p-value less than 0.05 were considered statistically significant.

To visually represent the results, enhanced volcano plot was used to show the fold change in log2 scales and p-values of these significant miRNAs. Dot plots were used to show the distributions of miRNAs in the e-cigarette and non-user groups. The pheatmap function in the pheatmap package in R was used to generate the heatmap of significant miRNAs. Additionally, a receiver operating characteristic (ROC) curve was constructed using the results of a logistic regression model with predictor variables including significant miRNAs and covariate, employing the rocit function in the ROCit package in R (R Core Team, 2019). The area under curve (AUC) of the ROC curve was calculated using the auc function in the pROC package in R. For molecular experiments the statistical analysis was performed using Student’s *t*-test. Data were analyzed using Graphpad prism software (version 7.02)

### Gene enrichment analysis

Predictions of gene targets for differentially expressed miRNAs were conducted using the EVmiRNA (http://bioinfo.life.hust.edu.cn/EVmiRNA) [53], miRDB (http://mirdb.org/) [54, 55], and ExoCarta (http://exocarta.org) [56–58] database. The target genes predicted from these databases were subsequently imported into the enrichrichment function within the enrichR package in R [59]. This facilitated gene enrichment analysis using the Gene Ontology (GO) 2021 and the Kyoto Encyclopedia of Genes and Genomes (KEGG) 2021 databases.

### Validation of the expression levels of miRNA has-125b-5p in Bronchial Epithelial Cells

#### Cell Culture and Transfection

Human bronchial epithelial cell (BEAS-2B, Cat#: CRL-9609) were procured from the American Type Culture Collection (ATCC). Beas2B cells were cultured in DMEM/F12K medium and supplemented with 5% FBS (Cat#: 10082147; Thermo Fisher Scientific) along with 1% Penicillin-Streptomycin-Glutamine (Cat#: 103–78016; Thermo Fisher Scientific) cells were maintained at 37°C and 5% CO_2_ in a humidified atmosphere. The normal/negative control (NC), miR-125b-5p mimics, and miR-125b-5p inhibitors were purchased from Thermo Fisher Scientific. Cells were transfected with the indicated miRNA mimics or NC using transfection reagent Lipofectamine RNAiMAX reagent (Invitrogen, Carlsbad, CA, United States) and then collected for the experiments. For miR-125b-5p treatment, Control (Lipofectamine 25μL), miRNA Mimic Negative Control (25ng/mL with nuclease free water), miR-125b-5p mimics (25ng/mL) and miR-125b-5p mimics (50ng/mL) were instilled to Beas2Bcells.

#### DNA Damage Assay

The DNA damage assay was carried out using the FlowCellect^™^ DNA Damage Histone H2A.X Dual Detection Kit (Cat#: FCCS025153, Merck) was used to evaluate the DNA damage according to the manufacturer’s instructions. The DNA damage was analyzed using the Luminex Millipore Guava EasyCyte ow cytometer.

## Results

### Demographic characteristics of the participants providing plasma samples

Table 1 displays the demographic characteristics of the participants who contributed plasma samples for analysis. Ethnicity, gender, and age groups are balanced between the exclusive e-cigarette user and the non-user group with similar frequency distributions. However, there is a significantly difference in the frequency distribution of race, with exclusive e-cigarette users having a higher proportion of White individuals than the non-user group (86% vs. 67%). Consequently, race was included as a covariate in the generalized linear model for miRNA differential analyses.

### Identification of differentially expressed miRNAs in exclusive adult electronic cigarette users

**Figure 1:**
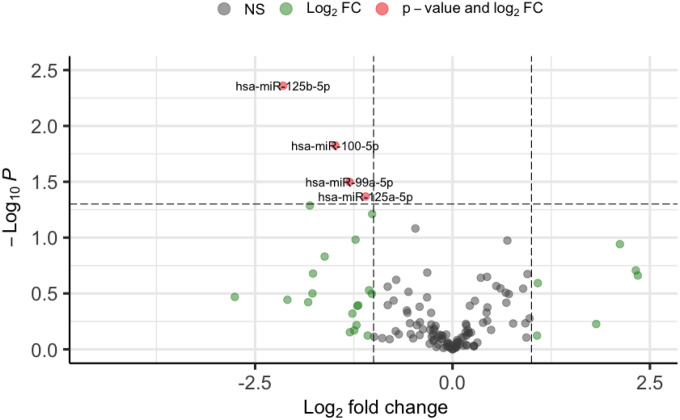
Volcano plot of significant miRNAs differentially expressed between exclusive e-cigarette users and non-users.

**Figure 2:**
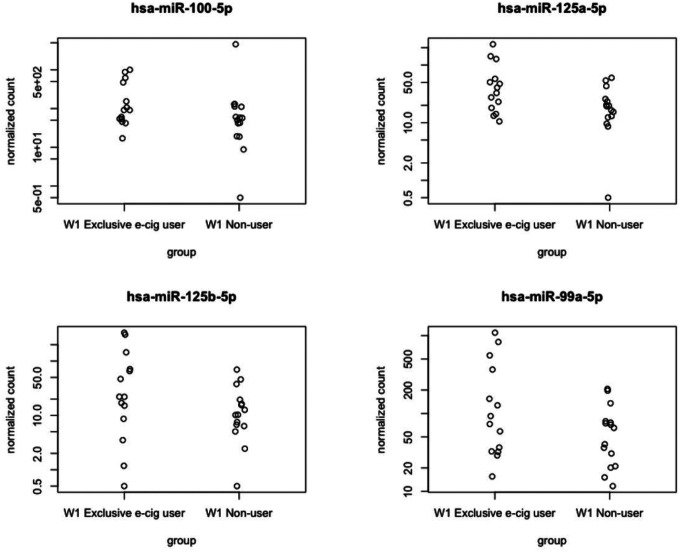
Distribution plot of significant miRNAs differentially expressed between exclusive e-cigarette users and non-users.

**Figure 3:**
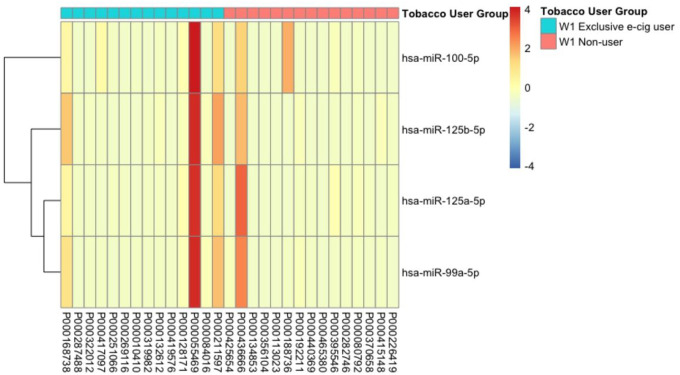
Heatmap of significant miRNAs differentially expressed between exclusive e-cigarette users and non-users.

Utilizing a cutoff of two times fold change and a *p*-value less than 0.05, we identified four significant miRNAs that were differentially expressed between exclusive e-cigarette users and non-users, as illustrated in the volcano plot (Fig. 1). Further examination of the distributions of these four significant miRNAs revealed significantly higher expression levels in the exclusive e-cigarette user group than the non-user group (Fig. 2). The heatmap of the four significant miRNAs, differentially expressed between exclusive e-cigarette users and non-users, highlighted their upregulation in the exclusive e-cigarette user group (Fig. 3). The identified significant miRNAs include hsa-miR-100–5p, hsa-miR-125a-5p, hsa-miR-125b-5p, and hsa-miR-99a-5p.

### Gene enrichment analysis of differentially expressed miRNAs in exosomes of exclusive adult electronic cigarette users

**Figure 4:**
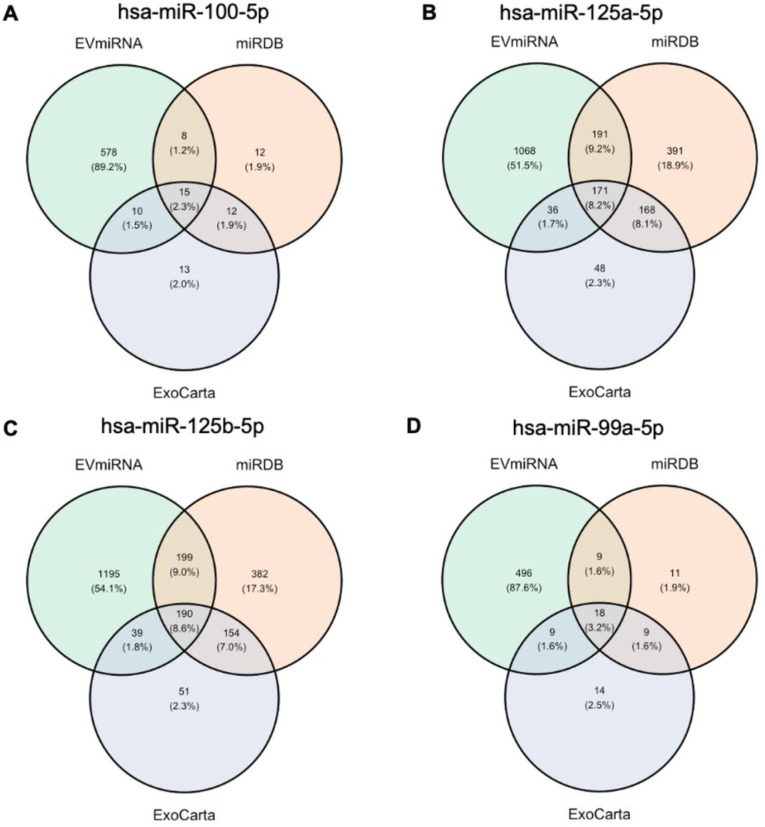
Target genes of significant miRNAs differentially expressed between exclusive e-cigarette users and non-users predicted by three different miRNA target prediction methods (EVmiRNA, miRDB, ExoCarta). A. has-miR-100–5p; B. has-miR-125a-5p; C. has-miR-125b-5p; D. has-miR-99a-5p.

The target genes of the four significantly upregulated miRNAs in exclusive e-cigarette users were predicted using the EVmiRNA (http://bioinfo.life.hust.edu.cn/EVmiRNA), miRDB (http://mirdb.org/), and ExoCarta (http://exocarta.org) databases. The Venn diagrams illustrate the overlap of target genes identified by all three methods for each significant miRNA (Fig. 4). Notably, variations of the number of target genes were observed among different miRNA target gene prediction methods, as well as variations of the number of target genes regulated by different miRNAs. EVmiRNA identified a greater number of gene targets than the other two methods. The union of target genes from all three methods was used for gene enrichment analysis.

**Figure 5:**
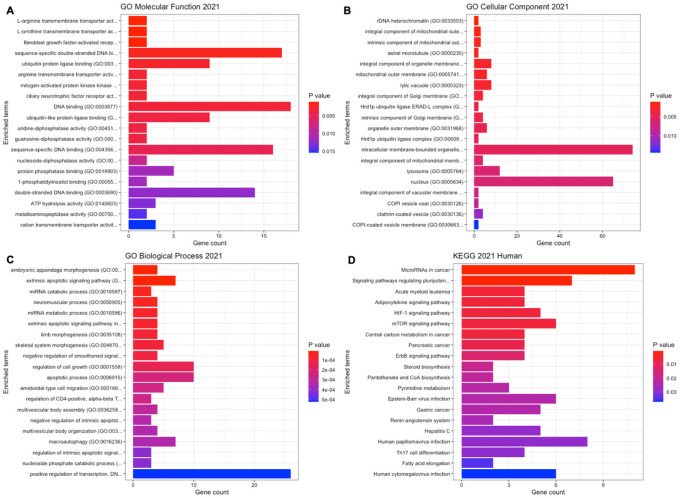
Gene enrichment analysis results using identified common target genes of significant miRNAs differentially expressed between exclusive e-cigarette users and non-users predicted by three different miRNA target prediction methods (EVmiRNA, miRDB, ExoCarta). A. GO Molecular Function 2021 database; B. GO Cellular Component 2021 database; C. GO Biological Process 2021 database; D. KEGG 2021 Human database.

Figure 5 showed the gene enrichment analysis results from the GO functional enrichment analysis of significant miRNA target genes and the systematic analysis of target gene functions using the KEGG 2021 Human database. The GO molecular function results highlighted the involvement of significant miRNA target genes in sequence-specific DNA binding, RNA-binding, ubiquitin protein ligase binding, and protein kinase binding. Genes related to intrinsic and integral component of mitochondrial and Golgi membrane were implicated in the GO cellular component analysis. The GO biological process revealed involvement in processes such as extrinsic apoptotic signaling pathway, miRNA catabolic process, miRNA metabolic process, embryonic appendage morphogenesis, and more. The KEGG analysis results identified target genes associated with various cancer pathways, including non-small cell lung cancer, small cell lung cancer, hepatocellular carcinoma, pancreatic cancer, Hippo signaling pathway, HIF-1 signaling pathway, p53 signaling pathway, and MAPK signaling pathway.

### Overexpression of miR-125b-5p Promote DNA damage in Bronchial Epithelial Cells

To elucidate the biological functions of miR-125b-5p in lung cells, we transfected the Beas2B cells with miR-125b-5p mimics and inhibitors. Initially, a wound-healing assay was performed. Overexpression of miR-125b-5p could effectively elicit a migratory response in the bronchial epithelial cells, 24 hrs after overexpression of miR-125b-5p promoted the migration and proliferation of the bronchial epithelial cells to the scratch area of bronchial epithelial cells, while miR-125b-5p knockdown showed effect. Additionally, we found that the overexpression of miR-125b-5p promoted DNA damage by expressing phosphorylation of gamma H2AX, whereas, inhibiting miR-125b-5p reduced this effect. We will be also studying the effect of overexpression of miR-125b-5p upon various e-cigarettes exposure in bronchial epithelial cells in future (Figure: 7). Taken together, these results suggest that miR-125b-5p might potentially exert the role of a suppressor gene in lung cells upon e-cigarette exposures.

### Receiver operating characteristic (ROC) curve for differentiation of exclusive e-cigarette users from non-users

**Figure 6:**
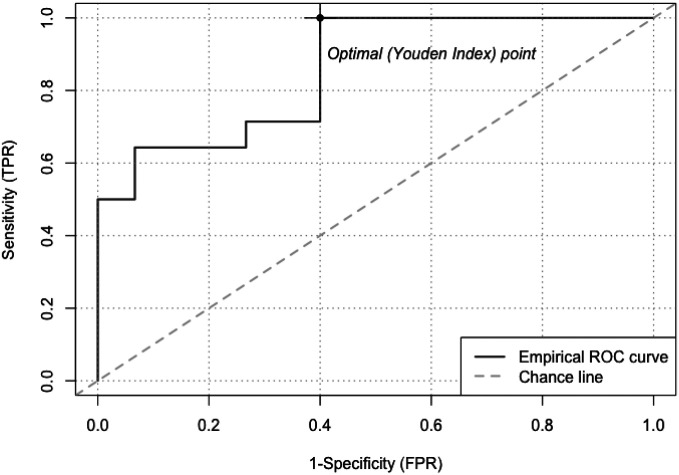
ROC curve of the four significant miRNAs differentially expressed between exclusive e-cigarette users and non-users and race for prediction of whether the participant is exclusive e-cigarette users or non-users. ROC curve showed that the sensitivity of the four miRNA markers and race for distinguishing non-users from exclusive e-cigarette users can reach 100% and the specificity can reach to 60% based on the optimal Youden Index point. The Area Under Curve (AUC) of the ROC is 0.8571, signifying excellent predictive capability when utilizing the four significant miRNAs in conjunction with race. TRP: true positive rate; FPR: false positive rate.

**Figure 7:**
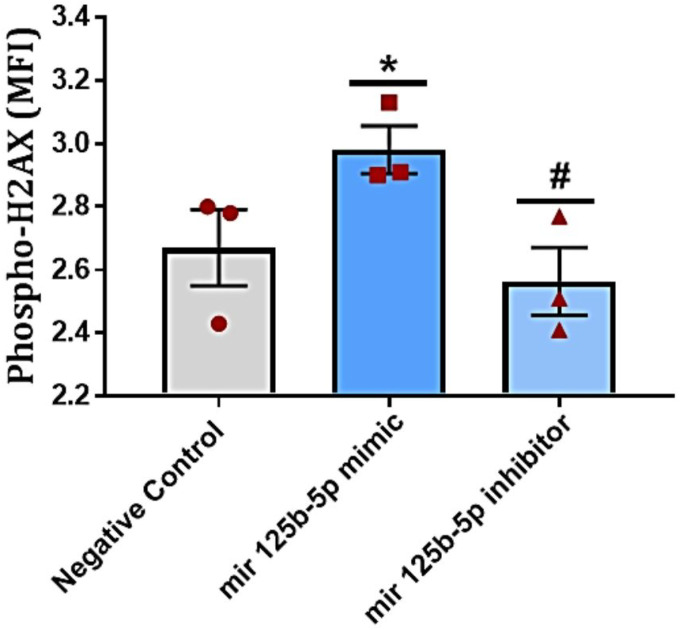
Overexpression of miR-125b-5p inhibited DNA damage in Bronchial Epithelial Cells: The Bronchial epithelial cells were seeded in six-well plates and transfected with miR-125b-5p mimic/inhibitors or the nonspecific control. 72 hrs Post-transfection cells were harvested for DNA damage assay. Overexpression of miR-125b-5p on DNA damage was examined by flow cytometry. Level of significance: *p<0.05 was considered significant compared to the negative control; #p<0.05 was considered significant compared to miR-125b-5p inhibitor treatment.

A ROC curve was constructed using the four significant miRNAs differentially expressed between exclusive e-cigarette users and non-users, along with race, to predict whether a participant falls into the category of exclusive e-cigarette users or non-users (Fig. 6). At the optimal Youden Index point, the sensitivity of the four significant miRNA markers and race reached 100%, with a specificity of 60%, effectively distinguishing exclusive e-cigarette users from non-users. The calculation of the Area Under Curve (AUC) yielded a value of 0.8571, indicating a highly reliable predictive capability for determining the e-cigarette use status using the four significant miRNAs and race.

## Discussion

Exosomal miRNAs play pivotal roles in intercellular communication and various biological processes [60, 61]. Dysregulation of exosomal miRNAs could link to tumorigenesis through regulation changes in central cellular process including cell proliferation, cell survival, and apoptosis. Current research has emphasized the potential of exosomal miRNAs as prognostic and diagnostic biomarkers for diseases [62]. While short-term adverse effects of e-cigarettes on human health have been well-documented, the long-term health effects of e-cigarette use remains unknown due to the relatively short period these products have been on the market [63, 64]. Few studies have examined the impact of e-cigarette use, particularly exclusive e-cigarette use, on exosomal miRNAs.

Using plasma samples from 30 participants in the PATH Wave 1 study, we investigated differences in exosomal miRNA expression levels between exclusive e-cigarette users and non-users. We identified four significant exosomal miRNAs (hsa-miR-100–5p, hsa-miR-125a-5p, hsa-miR-125b-5p, and hsa-miR-99a-5p) that were upregulated in exclusive e-cigarette users compared to non-users. Further gene enrichment analysis of predicted target genes regulated by these four significant miRNAs revealed their involvement in various cancer pathways. Our results suggest potential risks associated with exclusive e-cigarette use.

The involvement of hsa-miR-100–5p in numerous pathological processes associated with diseases has been documented. Overexpression of hsa-miR-100–5p in human ovarian endometriotic stromal cells has been linked to the promotion of invasion, contributing to the pathogenesis of endometriosis [65]. Additionally, hsa-miR-100–5p has been identified as an independent risk factor and a prognostic signature for patients with stomach adenocarcinoma [66]. Elevated expression levels of hsa-miR-100–5p have been associated with extracapsular extension and poorer survival in patients with oral squamous cell carcinoma compared to their normal counterparts [67]. In Alzheimer’s disease, hsa-miR-100–5p is known to regulate neuron survival by targeting the Mammalian Target of Rapamycin (mTOR) pathway, a central player in regulating many fundamental cell processes and a critical factor in tumor metabolism [68–70]. The upregulation of hsa-miR-100–5p in exclusive e-cigarette users, when compared to non-users, indicates potential elevated risks of various cancers.

The miRNA has-miR-125a-5p has been recognized as a tumor suppressor in various malignancies, including those affecting the breast, ovary, lung, and central nervous system [71, 72]. It plays a role in cell proliferation through cell cycle regulation and has potential as a therapeutic target for treating squamous cell carcinoma of the head and neck [71, 72]. Prior study has indicated that has-miR-125a-5p can induce apoptosis through a p53-dependent pathway in human lung cancer cells [73]. Furthermore, its associated with the pathological stage or lymph node metastasis in non-small cell lung cancer has been reported [74].

The miRNA hsa-miR-125b-5p has been identified as a regulator of inflammatory genes, targeting MAPKs and NF-kB signaling pathways in human osteoarthritic chondrocytes [75, 76]. Additionally, miR-125b-5p inhibits the expression of TNFR2, demonstrating immunosuppressive activity to enhance the antitumor efficacy in human colon adenocarcinoma patients [77]. In liver cancer, miR-125b-5p has been shown to inhibit cell proliferation, migration, and invasion [78]. The miRNA hsa-miR-99a-5p, recognized as a tumor suppressor in tumors like bladder cancer and breast cancer, suppresses cell proliferation, migration, and invasion [79]. Interestingly, a previous study found that the hsa-miR-99a-5p expression level in breast cancer tissues were significantly lower than healthy breast tissue, while the expression level in plasma samples were significantly higher in breast cancer patients than in healthy controls [80].

Although has-miR-125a-5p, miR-125b-5p, and hsa-miR-99a-5p are known tumor suppressors, it remains uncertain whether the upregulation of these exosomal miRNAs in plasma samples indicates an elevated risk in exclusive e-cigarette users. Our experimental validation showed that overexpression of miRNA hsa-miR-125b-5p can promote DNA damage in bronchial epithelia cells. Further research is necessary to evaluate the role of exosomal miRNAs in the association between exclusive e-cigarette use and adverse health effects.

Limited research has explored the impact of e-cigarette use on exosomal miRNA profiles. A prior study in 2019 investigated plasma exosomal miRNA expression levels, revealing a different set of miRNAs significantly differentially expressed between exclusive e-cigarette users and non-smokers [47]. This variation may stem from differences in e-liquid and e-cigarette devices. Our samples were collected during the PATH Wave 1 study from 2013 to 2014, aligning with the era of the first generation of e-cigarette devices [81]. During this period, most e-cigarettes were usually disposable single units containing natural nicotine in the e-liquid. In contrast, the predominant e-cigarette devices on the market in 2019 were fourth generation devices, largely disposable with nicotine salt in the e-liquid to mitigate nicotine harshness [81]. Given the large differences in e-liquid and e-cigarette devices, it is highly plausible that distinct exosomal miRNAs are influenced by e-cigarette use.

## Conclusions

In summary, our analysis of blood samples collected during the PATH Wave 1 study from 2013 to 2014 identified four plasma exosomal miRNAs including hsa-miR-100–5p, hsa-miR-125a-5p, hsa-miR-125b-5p, and hsa-miR-99a-5p. All four miRNAs were upregulated in exclusive e-cigarette users compared to non-users. Gene enrichment analysis of predicted target genes regulated by these four significant miRNAs uncovered numerous target genes associated with cancer-related pathways. Overexpression of miRNA hsa-miR-125b-5p can promote DNA damage in bronchial epithelia cells. These findings suggest a potential elevated risk of cancer among exclusive e-cigarette users. To validate these results, further investigations into the impact of exclusive e-cigarette use on plasma exosomal miRNAs and their correlation with cancer are warranted. The AUC of the ROC curve indicated a robust predictive ability of the four significant miRNAs when combined with race in distinguishing exclusive e-cigarette users from non-users.

## Figures and Tables

**Table 1:** Demographic characteristics of participants providing plasma samples.

Demographic Variable	Exclusive e-cigarette users n (%)	Non-users n (%)	P-value
Gender			1.000
Male	6 (40%)	6 (40%)	
Female	9 (60%)	9 (60%)	
Ethnicity			1.000
Hispanic	2 (13%)	1 (7%)	
Non-Hispanic	13 (87%)	13 (93%)	
Race			0.029
White alone	12 (86%)	10 (67%)	
Black alone	0 (0%)	5 (33%)	
Other	2 (14%)	0 (0%)	
Age group			1.000
18–24	8 (53%)	8 (53%)	
25+	7 (47%)	7 (47%)	

## Data Availability

The datasets generated and analyzed during the current study are available in the Gene Expression Omnibus (GEO) repository with access number GSE253603 [https://www.ncbi.nlm.nih.gov/geo/query/acc.cgi?acc=GSE253603]. The code used for the microRNA sequencing data analysis could be found from the GitHub website: https://github.com/DongmeiLi2017/plasma-exosomal-microRNA/tree/main.

## Abbreviations

E-cigarette: Electronic cigarette
EV: Extracellular vesicles
PATH: Population Assessment of Tobacco and Health
miRNA: microRNA
IRB: Institutional Review Board
SOP: Standard operating procedures
GO: Gene Ontology
KEGG: Kyoto Encyclopedia of Genes and Genomes
ROC: Receiver operating characteristic
AUC: Area under the curve
